# Co-Acquired Nanovirus and Geminivirus Exhibit a Contrasted Localization within Their Common Aphid Vector

**DOI:** 10.3390/v12030299

**Published:** 2020-03-10

**Authors:** Jérémy Di Mattia, Faustine Ryckebusch, Marie-Stéphanie Vernerey, Elodie Pirolles, Nicolas Sauvion, Michel Peterschmitt, Jean-Louis Zeddam, Stéphane Blanc

**Affiliations:** 1 UMR BGPI, Univ. Montpellier, INRAE, CIRAD, Montpellier SupAgro, 34398 Montpellier, France; jeremydimattia@gmail.com (J.D.M.); faustine.ryckebusch@cirad.fr (F.R.); marie-stephanie.vernerey@inra.fr (M.-S.V.); elodie.pirolles@inra.fr (E.P.); nicolas.sauvion@inra.fr (N.S.); michel.peterschmitt@cirad.fr (M.P.); Jean-Louis.Zeddam@ird.fr (J.-L.Z.); 2 UMR IPME, Univ. Montpellier, IRD, CIRAD, 34398 Montpellier, France

**Keywords:** fluorescent in situ hybridization, qPCR, leaf discs, Hemiptera, nanovirus, geminivirus, vector transmission

## Abstract

Single-stranded DNA (ssDNA) plant viruses belong to the families *Geminiviridae* and *Nanoviridae*. They are transmitted by Hemipteran insects in a circulative, mostly non-propagative, manner. While geminiviruses are transmitted by leafhoppers, treehoppers, whiteflies and aphids, nanoviruses are transmitted exclusively by aphids. Circulative transmission involves complex virus–vector interactions in which epithelial cells have to be crossed and defense mechanisms counteracted. Vector taxa are considered a relevant taxonomic criterion for virus classification, indicating that viruses can evolve specific interactions with their vectors. Thus, we predicted that, although nanoviruses and geminiviruses represent related viral families, they have evolved distinct interactions with their vector. This prediction is also supported by the non-structural Nuclear Shuttle Protein (NSP) that is involved in vector transmission in nanoviruses but has no similar function in geminiviruses. Thanks to the recent discovery of aphid-transmitted geminiviruses, this prediction could be tested for the geminivirus alfalfa leaf curl virus (ALCV) and the nanovirus faba bean necrotic stunt virus (FBNSV) in their common vector, *Aphis craccivora*. Estimations of viral load in midgut and head of aphids, precise localization of viral DNA in cells of insect vectors and host plants, and virus transmission tests revealed that the pathway of the two viruses across the body of their common vector differs both quantitatively and qualitatively.

## 1. Introduction

All ssDNA plant viruses belong to the families *Geminiviridae* and *Nanoviridae* and are transmitted by hemipteran vectors in a circulative, mostly non-propagative, manner [1,2], meaning that they persist and cycle within the body of their vectors without replication [3,4]. While nanoviruses are transmitted exclusively by insects of the family *Aphididae* (aphids) [5], geminiviruses are transmitted by three other hemipteran families: *Cicadellidae* (leafhoppers), *Membracidae* (treehoppers) and *Aleyrodidae* (whiteflies) [6,7,8]. Circulative transmission involves sophisticated virus–vector interactions in which viruses can cross insect epithelial membranes and escape intracellular degradation pathways and other insect immune defense mechanisms [9]. The fact that vector specificity can be used as a discriminating criterion for taxonomic assignment, particularly in the family *Geminiviridae* [10], reveals that vectors can structure virus populations via transmission specialization. While geminivirus-associated diseases have been studied for more than 100 years, a new genus had to be created recently in the family *Geminiviridae* (the genus *Capulavirus*) to accommodate the newly discovered aphid-transmitted species [11]. Alfalfa leaf curl virus was the first geminivirus for which aphid transmission was demonstrated, and its transmission by *Aphis craccivora* (Koch, 1854) was shown to be highly specific [12]. Unexpectedly, the transmission rate of ALCV per individual aphid was much lower than that of whitefly- and leafhopper-transmitted geminiviruses [13]. While ALCV was acquired and stably retained in midguts, the viral content in heads was relatively low and decreased over time, suggesting a low viral flow from the aphid body into the saliva [13]. Considering the late discovery of this genus, which may reflect a slow dissemination to, and within, cultivated hosts, these authors discussed the intriguing hypothesis that geminiviruses may not readily evolve virus–vector interactions that lead to high transmission efficiencies in aphids. From that, it is tempting to speculate that nanoviruses, which are all transmitted by aphids, have evolved virus–vector interactions that differ from those evolved by capulaviruses. In this case, different transmission routes, as well as different transmission rates, may be expected.

Nanoviruses and most geminiviruses are reported to be restricted to phloem tissues of their host plants [14], and species of both groups have been detected in the anterior midgut (AMG) and principal salivary glands (PSG) of their aphid vector [12,15,16,17]. Despite these similarities, striking differences also exist, and can be expected to have a significant impact on transmission. While the genome of capulaviruses consists of only one ssDNA circle, that of nanoviruses has a multipartite organization and consists of eight ssDNA circles, each encapsidated individually in a distinct virus particle. Moreover, while the CP is the only geminivirus protein reported to interact with insect vectors [18,19], the NSP of nanoviruses has been shown to act as a helper factor for transmission [20,21] and is mandatory for accumulation of virus particles within the cells of the aphid AMG [22]. Thus, the different genome architecture, and the use of a helper factor for the transmission of nanoviruses but not of geminiviruses, is consistent with potentially different adaptations to vectors in these two viral families.

Here, we explore this prediction by making use of the fact that the ALCV (capulavirus) and the FBNSV (nanovirus) both share a common host (broad bean) and a common aphid vector (*Aphis craccivora*). Our results show that the ALCV- and FBNSV-vector interactions are different based on several features. First, DNA accumulation of ALCV in the midgut is about 10 times higher than that of FBNSV. Second, in situations of co-infection, although ALCV has an apparent negative impact on FBNSV accumulation in aphids, the transmission rate of the latter remains 2–10 times higher than that of the former. Third, in spite of higher accumulation in aphids, ALCV is excreted in the saliva in much lower amounts than FBNSV. Finally, while both viruses accumulate in the same cells in the aphid AMG, they do not co-localize within these cells, suggesting that they use distinct pathways during transcytosis.

## 2. Materials and Methods 

### 2.1. Plant Material and Agro-Inoculation

Broad bean plants (*Vicia faba*, L. cv. “Séville”, Vilmorin, Paris, France) were maintained in growth chambers with 70% hygrometry, 26/20 °C day/night temperature and 13/11 h day/night photoperiod. The soil of each potted plant was sprayed with a 0.04% aqueous solution of Trigard 75 WP (ref: 24923, Syngeta^®^, Syngenta Global, Basel, Switzerland) to prevent development of sciarid flies.

The FBNSV agroinfectious clone was constructed earlier from an Ethiopian isolate [23]. It consists of eight distinct clones, each containing one of the eight FBNSV genome segments (C, M, N, R, S, U1, U2, U4) inserted as a head-to-tail dimer into the binary plasmid pBin19. Each of these eight plasmids were propagated into the COR308 strain of *Agrobacterium tumefaciens* and inoculated into plants as a mixture exactly as earlier described [24]. The agroinfectious clone of ALCV has also been previously described [11]. It consists of a tandem repeat of the whole genome sequence, inserted in the binary plasmid pCambia2300 and transformed in the C58 strain of *A. tumefaciens*, which can be inoculated into plants as a single agrobacterial culture [12]. FBNSV and ALCV clones were used to agroinoculate 10-day-old plantlets. The OD_600_ of agrobacteria cultures was monitored in order to inoculate the same amount (OD_600_ between 2 and 3 units) of cells for each clone in all experiments. This resulted in a number of bacterial cells being infiltrated into the plant, equal to 1× for mono-infection with ALCV, to 8× for mono-infection with FBNSV, to 9X for co-infection with both viruses. In one test (as indicated in the Results), FBNSV was inoculated by viruliferous aphids with an inoculation access period (IAP) of 2 days on 8-day-old plantlets, prior to agro-inoculation with ALCV.

Mono-infected or co-infected plants were all analyzed by qPCR (see below) to confirm the presence of ALCV and of each of the eight components of FBNSV.

### 2.2. Aphid Rearing

Individuals of *A. craccivora* came from a clonal colony established previously [13], and were reared on healthy broad bean plants maintained under a 24/18 °C day/night temperature and a 16/8 h day/night photoperiod. Viruliferous aphid cohorts were derived from this colony by placing apterous adult females on broad bean plants infected for approx. 30 days with FBNSV, ALCV or both. For transmission tests, these apterous females were transferred onto healthy plantlets after a 3-day acquisition access period (AAP). For in situ hybridization, the apterous females were removed following the 3-day AAP, and their larvae were left to develop on the infected plants for a further 10 days. This procedure yielded young viruliferous adults that had been in contact with the virus(es) for a longer period, (i) allowing abundant virus accumulation in the gut and salivary glands, and (ii) increasing the likelihood of observing the association of viruses with intracellular compartments in a form in which they are durably stored.

### 2.3. Plant and Insect Dissections

Veins from the upper leaves of infected plants were collected with an adhesive tape following a published protocol [25]. The veins were then fixed overnight at 4 °C under gentle stirring in embryo dishes containing 4% paraformaldehyde (PFA) and 0.2% Triton X-100 diluted in phosphate-buffered saline 1X pH 7.4 (PBS). Fixation was stopped by a 15-min incubation in 0.1 M glycine diluted in PBS followed by a washing step in PBS. To reduce chlorophyll autofluorescence, plant samples were incubated five times for 30 min each in a Carnoy solution containing 6 volumes of ethanol (EtOH), 3 volumes of chloroform and 1 volume of glacial acetic acid. This process was completed with a 1-h wash in absolute ethanol. Finally, fixed leaf veins were stored at 4 °C in PBS until use.

Between 30 and 50 viruliferous aphids, maintained for 10 days on an infected plant as described above, were placed for 48 h on healthy plants in order to clear the lumen from virus containing alimentary bolus. The AMG and PSG were then dissected in PBS by pulling the head off the abdomen with forceps. Organs were fixed in 4% PFA for 20 min and rinsed in 0.1 M glycine diluted in PBS for 15 min. After fixation, AMG and PSG were stored in PBS at 4 °C until use.

### 2.4. Fluorescent in Situ Hybridization (FISH)

Virus DNA-specific fluorescent probes were prepared by random priming with the BioPrime DNA labeling system kit (Invitrogen, Carlsbad, Calif, USA) as described [26]. Probes complementary to the ALCV CP gene incorporated Alexa Fluor 488-labeled dUTP (green), whereas those complementary to FBNSV incorporated Alexa Fluor 584-labeled dUTP (red). Probes were diluted 30 times before incubation with the samples. For FBNSV-infected plants, a mix of probes targeting the eight viral segments was used because infected cells do not necessarily contain all segments [27]. In aphids, U2- (green) and U4- (red) specific probes were used to co-localize FBNSV segments. In experiments assessing the co-localization of FBNSV and ALCV in midgut cells, only the U4-specific probe was used for FBNSV because it has previously been reported that AMG cells of viruliferous pea aphid, *Acyrthosiphon pisum*, always contain this (and likely all) FBNSV segments [22].

Prior to labeling, dissected insect organs or plant veins were incubated for 5 min at room temperature (RT) in 20 mM Tris-HCl hybridization buffer (pH 8) containing 0.9 M NaCl, 0.01% SDS and 30% formamide [28]. Samples were then incubated overnight at 37 °C with the diluted probes in hybridization buffer, and then rinsed 3 × 5 min at room temperature in the hybridization buffer followed by two final rinses for 10 min each in PBS.

Samples were mounted on microscope slides in Vectashield^®^ antifade mounting medium (Vector Laboratories, Burlingame, CA, USA) containing 1.5 µg·mL^−1^ of DAPI [26]. Observations were performed with a LSM700 confocal microscope (Zeiss, Oberkochen, Germany) and all images were taken at a resolution of 1024 × 1024 pixels with a ×63 objective. 

### 2.5. Viral DNA Extraction

For plants, three leaf discs of 0.6 cm diameter were squashed onto Whatman paper from the upper leaf-level of infected broad bean plants. Each Whatman paper disc was then deposited individually onto the filter of 200 µL micropipette tips and 100 µL of modified Edwards buffer (200 mM Tris-HCl pH 7.5, 25 mM EDTA, 250 mM NaCl, 0.5% SDS, 1% PVP40, 0.2% ascorbic acid) were added. For insects, groups of five heads or abdomens were ground with a plastic pestle in a 1.5 mL Eppendorf tube containing 100 µL of modified Edwards buffer (without ascorbic acid). Samples were similarly deposited onto the filter of 200 µL tips, and the homogenate was centrifuged directly into a PCR plate placed underneath, at 5000 *g* for 15 s. Finally, the DNA was precipitated with isopropanol, washed once with 70% EtOH and resuspended in 50 µL of distilled water before further processing [12]. 

### 2.6. Quantitative Real-Time PCR (qPCR) Detection

ALCV and FBNSV DNAs were qPCR-detected and quantified in a LightCycler 480 thermocycler (Roche, Indianapolis, Ind, USA) using 2 µL total DNA extracts diluted 10-fold in H_2_O. The LightCycler FastStart DNA Master Plus SYBR Green I kit (Roche) was used according to the manufacturer’s instructions with 5 µL of the 2X qPCR Mastermix, 0.3–0.6 μM final primers (0.3 µM final for segments C, M and S of FBNSV, 0.5 µM final for the other segments of FBNSV and 0.6 µM final for ALCV), complemented with H_2_O to obtain 8 µL of final mix for 2 µL of DNA sample as matrix (10 µL total). The specific primers of FBNSV and ALCV used for the qPCR were described in references [12,24], respectively. Forty qPCR cycles of 95 °C for 10 s, 60 °C for 10 s and 72 °C for 10 s were applied to the samples. All samples were analyzed with two technical replicates.

Post-PCR data analyses were carried out as described [29]. The accumulation of ALCV is expressed as the number of copies of monopartite viral genomes. Accumulation of FBNSV is expressed as the sum of the number of copies of the eight genome segments.

### 2.7. Transmission Experiments

Apterous adult aphids were given a 3-day AAP on infected broad bean plants followed by a 3-day IAP on 10-day-old healthy broad bean plants with 10 individual aphids per test plant. IAP was stopped with a spray of insecticide (Pirimor–Certis^®^, Marguerittes, France), 1 g·L^−1^ in water). At 21 days after the beginning of the IAP, the presence of FBNSV was checked visually by inspection of symptoms, and that of ALCV by qPCR. All transmission tests were carried out in the containment room where aphid colonies are maintained, with the exception of the fifth test, which was carried out in a containment chamber with 16 h light at 26 °C, and 8 h dark at 24 °C.

### 2.8. Leaf-Disc Inoculation Assay and Nested-PCR

Leaf-disc inoculation assays were performed to estimate the amount of viral DNA inoculated by aphids into plants. Leaf-discs of 2.5 cm diameter were maintained in 6-well cell culture plates, one disc per well, on 1.5% agarose + 2% Na_2_SO_3_ in PBS to limit drying and oxidation. A first experiment was performed under environmental conditions of the fifth transmission test. A second experiment was performed under the standard conditions of the aphid growth chambers. Aphids were given a 3-day AAP on infected plants and then transferred onto healthy plants for 3 days to clear the gut lumen of virus material and thus prevent surface leaf-disc contamination by viruliferous honeydew. These “purged” aphids were transferred onto a first series of leaf-discs (10 individuals per disc) for an IAP of 24 h, and then moved to a second series of leaf discs for an additional IAP of 48 h. After aphid removal, the leaf discs were double washed in a detergent solution (Magister^®^, company, Epernay, France)—detergent manual plunge—1:20 dilution in H_2_O) followed by two rinses in H_2_O to remove residual viral contamination by feces/honeydew. Between each wash, leaf-discs were further cleaned with a smooth paintbrush. Finally, to prepare the DNA extraction step, leaf-discs were placed in 2 mL microtubes (Sarstedt, Nümbrecht, Germany) with two metal beads of 3 mm diameter and Fontainebleau sand. Micro-tubes containing the samples were immerged in liquid nitrogen for flash freezing and stored at −80 °C if required. A volume of 750 µL of modified Edwards buffer was added in each tube where the frozen leaf discs were ground by shaking four to six times each for 20 s to obtain a homogeneous solution (FastPrep^®^-24, MP Biomedicals^®^ Instrument IIIkirch-Graffenstaden, France). Total DNA was extracted using the Purelink^TM^ plant total DNA purification kit (Invitrogen) and qPCR was carried out on 100-fold diluted samples.

For nested-PCR experiments, the extracted DNA was purified a second time with the same Purelink^®^ Plant total DNA kit (Invitrogen), and each sample was tested in triplicate to control the presence of ALCV. The GoTaq^®^ G2 DNA polymerase (Promega, Madison, WI, USA) was used according to the manufacturer’s instructions, using 1 µL of the double-purified DNA, 0.25 µL polymerase, 10 µL of 5× green GoTaq^®^ reaction buffer, 38.25 µL H_2_O, 0.5 µL dNTP and 0.5 µL of both forward and reverse primers at 10 mM. The primer pair used for the first PCR was: CP_ALCV_185-F ^5′^TGGAATATTGTGCTGCTTGG^3′^ (position 185 on the virus genome; forward orientation) and: CP_ALCV_1025-R ^5′^GTGGTCTATTTCAGCAGTTGC^3′^ (position 1025; reverse orientation). The pair of primers for the second PCR was CP_ALCV_620-F ^5′^GAAGAGGGCGAAAACGACAG^3′^ (position 620 on the genome; forward) and CP_ALCV_969-R ^5′^ATTTTGGGACTTGTGCTCCA^3′^ (position 969; reverse). Each PCR was performed with 40 cycles of amplification: 98 °C for 2 min, 98 °C for 20 s, 58 °C for 20 s, 72 °C for 1 min and 72 °C for 5 min. 

### 2.9. Statistical Analysis

All statistical analyses were conducted with the package *stats* of the R software package, v3.5.0 [30]. As also indicated in the figure legends, boxplots display DNA quantification in source plants performed on *n* = 9, *n* = 11 and *n* = 19 plants infected, respectively, with FBNSV, ALCV and both viruses. Differences in the amounts of each virus DNA in abdomens and heads were tested with a Tukey HSD test on the transformed log10 values. ANOVA were performed with the virus (ALCV or FBNSV), the “organ” (abdomen or head) and the type of infection (mono- or co-infection) as the tested variables. 

## 3. Results

### 3.1. FBNSV and ALCV Can Co-Infect Plants and Cells within These Plants

**Figure 1:**
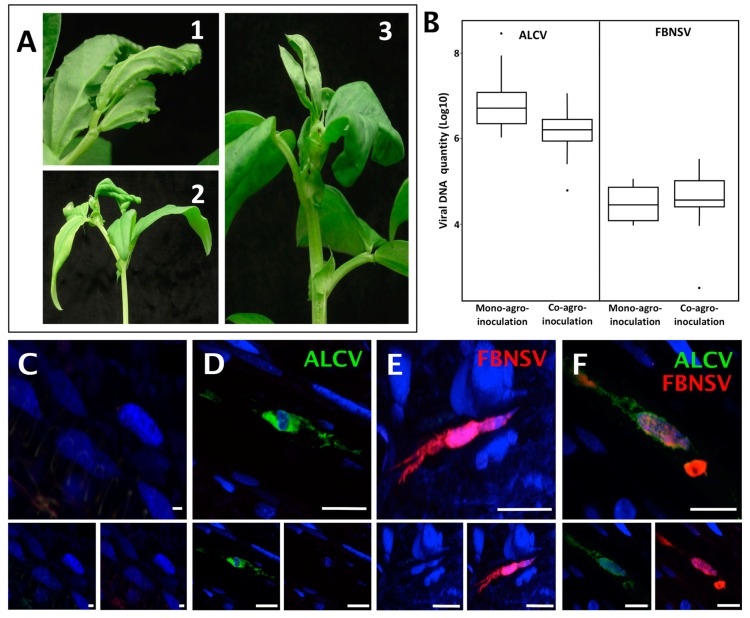
Localization of FBNSV and ALCV DNA in broad bean host plants. Visualization of ALCV symptoms (**A1**) and FBNSV symptoms (**A2**) on late stage of infection on broad bean plants, where symptoms are easier to see, and ALCV + FBNSV symptoms (**A3**) on early stage of infection, when over-curling of leaves due to FBNSV do not yet totally mask ALCV-associated vein swelling. (**B**) Box-plots of viral DNA amounts (log10 of the copy number, see Materials and Methods) of each virus in mono- and co-agro-inoculated broad bean plants. The box-plot on the mono- and co-infected plants represent *n* = 9 for FBNSV, *n* = 11 for ALCV and *n* = 15 for co-infected plants, respectively. C to F: Localization of ALCV DNA (green probe) and FBNSV DNA (8 segments labeled, red probe) in phloem tissues of broad bean plants either mock infected (**C**), or co-infected with the two viruses (**D**–**F**). Most cells contain either ALCV (**D**) or FBNSV (**E**) alone, but they can also occasionally be co-infected (**F**). Top panel in C to D are images with merged color channels and the corresponding split channels are at the bottom left (green) and right (red). Each image corresponds to maximum intensity projections. The scale bar of each image represents 25 µm. Cell nuclei are DAPI blue-stained.

Both FBNSV and ALCV can individually infect broad bean [11,31]. To investigate and compare the mechanisms of their transmission, we first evaluated whether they can co-infect the same individual host plants, from which they could be concomitantly/simultaneously acquired by the insect vector. When agro-inoculated alone, the mean infection rate was 41%^(+/−33%)^ for FBNSV and 93%^(+/−12%)^ for ALCV (percent calculated from five experiments with 96 plants per experiment for FBNSV and 24 plants per experiment for ALCV). When co-agroinoculated, 9%^(+/−2%)^ of the plants were co-infected, while FBNSV was found in 31%^(+/−20%)^ and ALCV in 66%^(+/−18%)^ (percent calculated from five experiments with 48–72 agro-inoculated plants per experiment). This slightly lower infection rate of both FBNSV and ALCV when co-agroinoculated could stem from the trivial fact that the total amount of bacteria agro-inoculated per plant differs greatly in mono- and co-inoculation (see Materials and Methods). We did not investigate this specific point further because our primary goal was to obtain co-infected plants for further characterization. More important is the observation of identical symptoms in mono- and co-infected plants (Figure 1A) and the absence of interference between the two viruses for accumulation in plant tissues. Indeed, at 21 days post infection (dpi), both viruses accumulated similarly in mono- and co- infections (*p*-value = 0.15 and 0.40 for FBNSV and ALCV, respectively), with ALCV reaching a viral load about 100 times that of FBNSV in all cases (Figure 1B).

The localization of ALCV and FBNSV was determined in co-infected plant tissues by in situ hybridization. ALCV and FBNSV DNAs were both detected in phloem vessels, most likely in companion cells, but only in some infected areas, with a large proportion of the observed cells being void of viruses. Because the two viruses appear to infect only a fraction of the susceptible cells—a phenomenon already reported for FBNSV [27]—it follows that they are often found alone (Figure 1D–E) and only occasionally together (Figure 1F). 

Overall, our results demonstrate not only that FBNSV and ALCV can co-exist in the same host plant, where they accumulate as if infecting alone, but also that these viruses can co-exist in the same individual cells of these plants, suggesting little or no interference during the colonization process. Plants in which both viruses co-circulate in the sieve elements can act as source plants for concomitant virus acquisition by the aphid vector.

### 3.2. FBNSV and ALCV Are Co-Acquired from Co-Infected Plants

**Figure 2:**
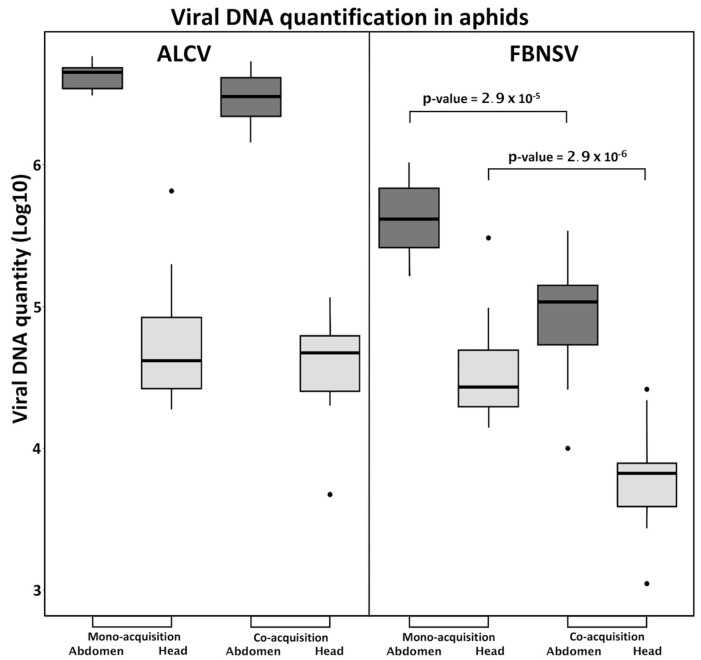
Accumulation of FBNSV and ALCV in aphid vectors. The box-plots represent the amounts of viral DNA (log10 of copy number) of ALCV and FBNSV in the abdomens (dark grey) and heads (light grey) of *A. craccivora*. The box-plots are from pools of five abdomens or heads with *n* = 11 for monoinfection and *n* = 15 for coinfection. *p*-values given result from testing the differences in FBNSV DNA amounts in heads or abdomens of aphids that have acquired FBNSV from mono-infected versus co-infected plants.

The circulative, non-propagative transmission of ALCV and FBNSV in aphids was documented recently, in independent studies, and the route of the two viruses within the vector appears strikingly similar [12,22]. Here, to test for potential interference during virus intake, we measured the viral load in aphid vectors after a 3-day AAP on mono- or co-infected plants. When acquired from mono-infected plants, ALCV accumulated in the abdomen to higher levels than FBNSV (Figure 2), likely reflecting its higher accumulation within co-infected plants (Figure 1B). Interestingly, this discrepancy was no longer observed in the heads, suggesting a lower flow of ALCV than of FBNSV between the aphid gut and salivary glands.

When acquired from co-infected plants, FBNSV and ALCV were both detected in heads and abdomens, indicating that the acquisition and transit from gut to salivary glands of one virus does not preclude that of the other (Figure 2). When considering the amounts accumulated, however, ALCV was not affected by co-infection, whereas FBNSV decreased by about one order of magnitude, both in the abdomen, and likely also in the head. This reduced accumulation of FBNSV in the presence of ALCV is discussed further below. 

### 3.3. FBNSV and ALCV Accumulate in Distinct Cytoplasmic Aggregates in Aphid Gut Cells 

**Figure 3:**
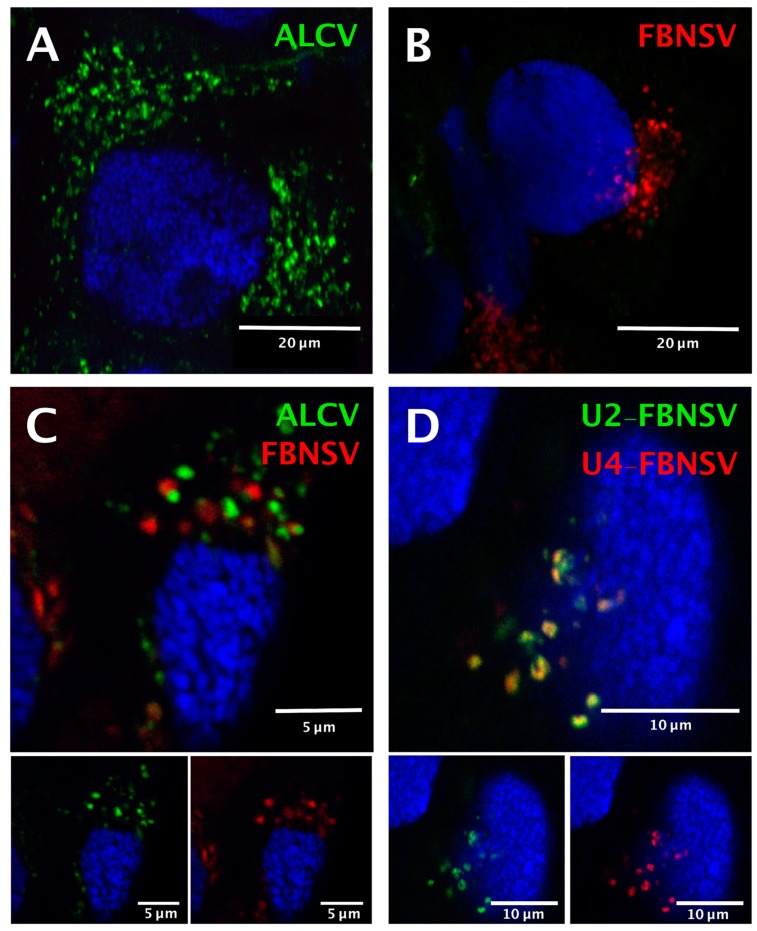
Localization of ALCV and FBNSV DNA in anterior midgut cells of *A. craccivora*. Co-labeling of ALCV (green) and FBNSV (8 segments probes, red) in aphids fed on plants infected with ALCV alone (**A**), FBNSV alone (**B**), or with both viruses (**C**). Co-labeling of the two FBNSV segments U2 (green) and U4 (red) in aphids fed on plants co-infected by FBNSV and ALCV (**D**). Top panels in C and D are images with merged color channels, and the corresponding split channels are at the bottom left (green) and right (red). Images A and B correspond to maximum intensity projections and images C and D to single optical sections. Cell nuclei are blue-stained with DAPI.

The two viruses were localized precisely in cells of the AMG of individual aphids that fed on co-infected plants, as reported earlier for aphids fed on plants singly infected with either FBNSV [22] or ALCV [12]. Both viruses were detected in the majority of individual AMG cells, indicating that they are not mutually exclusive (Figure 3).

Because the insects were killed and processed for FISH analysis 2 days after the end of the AAP, after a “purging” period on healthy plants, detection of the two viruses confirms that neither entry nor persistence is hindered by co-infection. The intra-cellular FBNSV and ALCV aggregates (fluorescent foci) that were detected in the same individual gut cells were similar in size and shape, and were distributed predominantly around the nucleus (Figure 3C), just as in aphids fed on singly infected plants (Figure 3A,B). Remarkably, each aggregate was strictly specific to either ALCV or FBNSV, and the respective fluorescent labeling never overlapped (Figure 3C).

As a control for this observation, we made use of the fact that, in aphids fed on co-infected plants, the ingested sap contains ALCV particles as well as FBNSV particles representing encapsidation of individual distinct segments. While particles of the two viral species apparently segregate upon internalization in AMG cells, particles of FBNSV containing distinct segments do not, as recently demonstrated in the aphid A. pisum [22]. In the same A. craccivora cohort as that used to localize ALCV and FBNSV simultaneously (Figure 3C), we also specifically labeled two distinct segments of FBNSV, namely U2 and U4, and confirmed that they enter cells and accumulate together (Figure 3D). This result elegantly illustrates that when the aphid vector ingests a fluid containing a mixture of virions of the two species (the sap of co-infected plants), FBNSV particles all follow the same intracellular pathway, whatever segment they contain, and that this pathway is different from that followed by ALCV particles.

### 3.4. FBNSV and ALCV Are Both Released Together with the Aphid Saliva

The FBNSV content of *A. craccivora* individuals fed on co-infected plants was about 10 times lower than that of those fed on mono-infected plants (Figure 2), including the heads, and thus likely the salivary glands. To test the potential effect of this lower FBNSV content on transmission, we first carried out an inoculation assay on broad bean leaf discs, as described in Section 2. FBNSV DNA was readily detected by qPCR after a 24-h IAP in most leaf discs previously loaded with aphids fed on mono- or co-infected plants (Table 1). 

In contrast, ALCV DNA could be detected by qPCR only after a longer IAP period of 48 h, in only 2 leaf-discs out of 38 tested in this condition, and only when aphids had previously acquired the virus from mono-infected plants. To confirm this unexpected low rate of ALCV inoculation with respect to its relatively high accumulation in aphid heads (Figure 2), the presence of ALCV DNA was further assessed by nested-PCR. With this more sensitive technique, all leaf-discs proved negative after a 24-h IAP, while a few gave a positive signal after the longer 48-h IAP, with aphids fed on either mono-infected (9/38) or co-infected (5/41) plants (Table 1).

In the case of co-infection, the five leaf-discs where ALCV DNA could be detected also contained FBNSV DNA (Table 1), showing that the two viruses can be secreted in the saliva of the same individual aphid. Interestingly, a Chi^2^ test showed no statistical difference in the presence of ALCV or FBNSV DNA in leaf-discs whether aphids had previously been fed on mono- or co-infected plants (*p*-value = 0.24 and 1, respectively), revealing no interference between these viruses for inoculation success.

### 3.5. FBNSV Antagonizes ALCV at the Onset of Infection after Co-Transmission by Aphids

Finally, beyond release into leaf-discs, we tested whether ALCV can be transmitted effectively to plants when it is co-inoculated together with FBNSV by the aphid vector. Transmission tests from mono- and co-infected plants showed that FBNSV is always transmitted much more efficiently than ALCV (Table 2), consistent with their respective release into leaf discs (Table 1). Nevertheless, the ALCV transmission rate from mono-infected plants was significantly higher than that from co-infected plants (Table 2), which may be interpreted as a negative interference of FBNSV. Intriguingly, however, in the only two plants infected with ALCV in co-transmission experiments, FBNSV was not present. It is thus possible to imagine that FBNSV might only affect ALCV within the newly inoculated plant solely at the onset of infection, but not during the whole cycle within the vector.

## 4. Discussion

Numerous studies and reviews have cited the common features of the two families *Geminiviridae* and *Nanoviridae* to assume similar mechanisms of transmission for all ssDNA plant viruses [1,2,32,33]. Because no gemini- and nanovirus members were known to share a common vector species, this assumption was difficult to confirm. The discovery of ALCV and its aphid vector opened, for the first time, the opportunity to compare the life cycle of a geminivirus (ALCV) and a nanovirus (FBNSV) within a common host plant and its common insect vector.

### 4.1. Co-Infection of Plants by a Nanovirus and a Geminivirus

Co-infections of plants are common, if not the rule, in nature [34]. Yet, to our knowledge, the co-acquisition and inoculation of ALCV and FBNSV by aphids is the first report of co-circulation within the same insect vector for circulative viruses that belong to different families. However, the frequent co-infection of whitefly-transmitted geminiviruses and nanovirus-like alphasatellites (formerly named DNA1) suggests that co-infection of geminiviruses and nanoviruses is a widespread phenomenon [35,36]. Alphasatellites have the highly conserved nanovirus-like sequence motif TAGTATTAC and a replication associated gene (Rep) homologous to that of nanoviruses, indicating that these satellites may have evolved from components of nanoviruses, perhaps from historical occurrences of co-infection [37]. The experimental co-infection of ALCV and FBNSV described in the present paper provides direct proof that gemini- and nano-viruses can co-infect plants. Moreover, the detection of co-infected cells within these plants is consistent with inter-family recombination, as illustrated by the defective def19 DNA molecule found associated with a begomovirus [35]. In our experimental conditions, co-infection was possible only via co-agroinoculation. Co-infection by aphid vectors, although highly probable, remains to be confirmed. 

### 4.2. Possible Interference between ALCV and FBNSV

The accumulation of FBNSV DNA in the abdomens and heads of aphid vectors was lower when FBNSV was co-acquired with ALCV. Several explanations can be proposed: (i) For unknown reasons, the accumulation of FBNSV could be lower in the sieve elements of plants co-infected with ALCV; a comparative quantification of FBNSV in total leaf tissues (as here) and phloem exudates could confirm or refute this hypothesis. (ii) The presence of ALCV could elicit insect defense mechanisms (e.g., autophagy) as shown for other geminiviruses [9], which might differentially affect FBNSV and ALCV. (iii) The two viruses could compete for entry and/or accumulation in aphid AMG cells [38], and ALCV could partially outcompete FBNSV. The latter seems improbable, however, since the two viruses seemingly use distinct entry and accumulation routes (see below).

The transmission results were quite unexpected. While the accumulation of ALCV DNA in the midgut is about 10 times higher than that of FBNSV, the latter is transmitted 2–10 times more efficiently than ALCV, irrespective of infection status. Unexpectedly, the negative impact of ALCV on FBNSV accumulation did not impair its excretion into leaf discs or its transmission. Also unexpected was the observation that, while the accumulation of ALCV is not affected by the presence of FBNSV, its transmission rate appears reduced. This cannot be explained by a deficiency in ALCV excretion, because no significant differences could be detected when comparing mono- and co-infection. Instead, we hypothesize that FBNSV might block an early step in ALCV infection in the host plant after co-inoculation by aphids. In support of this hypothesis, we observed that when FBNSV was inoculated earlier and in larger amounts in the leaf discs, test plants in co-transmission experiments became infected predominantly with FBNSV alone, with ALCV infecting only those rare plants where FBNSV infection failed.

### 4.3. Distinct Intracellular Pathways for Nanovirus and Capulavirus in the Insect Vector

Although ALCV and FBNSV infect the same cells of the AMG, they do not co-localize in the cytoplasm of these cells. This original observation is consistent with the prediction that geminiviruses and nanoviruses may follow distinct subcellular pathways across their insect vectors. Unfortunately, due to limited sensitivity and/or accessibility, viral DNAs were not detectable in PSG under our experimental conditions, as reported earlier [12,22].

The absence of intracellular colocalization of ALCV and FBNSV DNAs may reflect different transmembrane receptors that may, in turn, guide these viruses into distinct routes across the cells. The absence of homology between the proteins that are reported to bind putative insect receptors for geminiviruses (CP) [1,10,39] and nanoviruses (NSP) [20,21,22] is consistent with this interpretation. Other compelling evidence in support of this interpretation is that ALCV is specifically transmitted only by certain populations of *A. craccivora* [12], whereas FBNSV is more widely transmitted by *A. pisum*, *Myzus persicae* and *A. craccivora* [40]. Although several studies have analyzed insect–virus interactions in the two viral families [41,42,43], no receptor(s) have been identified so far. Thus, further effort is needed to confirm and provide experimental support to molecular models of the different transmission pathways adopted by nano- and geminiviruses as suggested here from comparison of FBNSV and ALCV.

## 5. Conclusions

Our results demonstrate that the transmission pathways of ALCV and FBNSV are qualitatively and quantitatively different in the vector *A. craccivora*. This original result is consistent with the prediction that geminiviruses and nanoviruses have evolved different virus–vector interactions. The low rate of ALCV transmission by aphids reported earlier [13] is confirmed here by comparison with a nanovirus under the same experimental conditions and with the same *A. craccivora* cohort. Altogether, these results indicate that ALCV has not evolved virus–aphid interactions that readily lead to high transmission efficiencies. It is remarkable that the eight components of the nanovirus were conveyed from plant to plant by the aphid vector more easily than the unique capulavirus component. 

## Figures and Tables

**Table 1:** FBNSV and ALCV DNA detection in broad bean leaf discs after aphid inoculation.

	Detection	qPCR	Nested-PCR	qPCR	Nested-PCR
		24 h		48 h	
Mono-inoculation	ALCV ^a^	0/25	0/25	2/38	9/38
	FBNSV ^b^	6/6	nt	13/14	nt
Co-inoculation	ALCV ^c^	0/26	0/26	0/41	5/41
	FBNSV ^d^	24/26	nt	38/41	nt
	ALCV + FBNSV ^e^	0/26		5/41	

^a^ Number of ALCV-positive leaf discs tested by qPCR or nested-PCR after IAP of 24 or 48 h by aphids from mono-infected plants. ^b^ Number of FBNSV-positive leaf discs tested by qPCR after IAP of 24 or 48 h by aphids from mono-infected plants. ^c^ Number of ALCV-positive leaf discs tested by qPCR or nested-PCR after IAP of 24 or 48 h by aphids from co-infected plants. ^d^ Number of FBNSV-positive leaf discs tested by qPCR after IAP of 24 or 48 h by aphids from co-infected plants. ^e^ Number of ALCV-(tested by nested-PCR) and FBNSV-positive (tested by qPCR) leaf discs after IAP of 24 or 48 h by aphids from co-infected plants.

**Table 2:** Co-transmission testing of FBNSV and ALCV.

Source Plants	Detection	Transmission Rate	
		Test 1-4	Test 5
Mono-inoculation	ALCV ^a^	4/30	16/28
	FBNSV ^b^	19/20	5/7
Co-inoculation	ALCV ^c^	0/54	2/29
	FBNSV ^d^	49/54	27/29
	ALCV + FBNSV ^e^	0/54	0/29

^a^ Number of plants infected after transmission of ALCV from mono-infected plants. ^b^ Number of plants infected after transmission of FBNSV from mono-infected plants. ^c^ Number of plants infected by ALCV after transmission from co-infected plants. ^d^ Number of plants infected by FBNSV after transmission from co-infected plants. ^e^ Number of plants infected by ALCV and FBNSV after transmission from co-infected plants.
